# Pre-stimulus Alpha Oscillations and Inter-subject Variability of Motor Evoked Potentials in Single- and Paired-Pulse TMS Paradigms

**DOI:** 10.3389/fnhum.2016.00504

**Published:** 2016-10-07

**Authors:** Zafer Iscan, Maria Nazarova, Tommaso Fedele, Evgeny Blagovechtchenski, Vadim V. Nikulin

**Affiliations:** ^1^Centre for Cognition and Decision Making, National Research University Higher School of EconomicsMoscow, Russia; ^2^Research Center of NeurologyMoscow, Russia; ^3^Department of Neurosurgery, University Hospital of Zurich, University of ZurichZurich, Switzerland; ^4^Laboratory of Neuroscience and Molecular Pharmacology, Institute of Translational Biomedicine, Saint Petersburg State UniversitySaint Petersburg, Russia; ^5^Neurophysics Group, Department of Neurology, Charité – University Medicine BerlinBerlin, Germany

**Keywords:** brain stimulation, variability, paired-pulse, motor evoked potentials, electroencephalography, oscillations

## Abstract

Inter- and intra-subject variability of the motor evoked potentials (MEPs) to TMS is a well-known phenomenon. Although a possible link between this variability and ongoing brain oscillations was demonstrated, the results of the studies are not consistent with each other. Exploring this topic further is important since the modulation of MEPs provides unique possibility to relate oscillatory cortical phenomena to the state of the motor cortex probed with TMS. Given that alpha oscillations were shown to reflect cortical excitability, we hypothesized that their power and variability might explain the modulation of subject-specific MEPs to single- and paired-pulse TMS (spTMS, ppTMS, respectively). Neuronal activity was recorded with multichannel electroencephalogram. We used spTMS and two ppTMS conditions: intracortical facilitation (ICF) and short-interval intracortical inhibition (SICI). Spearman correlations were calculated within and across subjects between MEPs and the pre-stimulus power of alpha oscillations in low (8–10 Hz) and high (10–12 Hz) frequency bands. Coefficient of quartile variation was used to measure variability. Across-subject analysis revealed no difference in the pre-stimulus alpha power among the TMS conditions. However, the variability of high-alpha power in spTMS condition was larger than in the SICI condition. In ICF condition pre-stimulus high-alpha power variability correlated positively with MEP amplitude variability. No correlation has been observed between the pre-stimulus alpha power and MEP responses in any of the conditions. Our results show that the variability of the alpha oscillations can be more predictive of TMS effects than the commonly used power of oscillations and we provide further support for the dissociation of high and low-alpha bands in predicting responses produced by the stimulation of the motor cortex.

## Introduction

While single-pulse TMS (spTMS) allows studying corticospinal excitability (Kujirai et al., 1993; Nakamura et al., 1997; Chen et al., 1998; Sanger et al., 2001; Chen, 2004), paired-pulse TMS (ppTMS) (Kujirai et al., 1993) provides a possibility to gain additional information about intracortical inhibitory/excitatory processes. One of the intensely discussed issues in sp- and ppTMS research, strongly affecting its sensitivity, and reproducibility (Goldsworthy et al., 2016) is a large trial-to-trial variability of the responses to TMS including motor evoked potentials (MEPs; Ellaway et al., 1998; Zarkowski et al., 2006; Mitchell et al., 2007; Sauseng et al., 2009; Jung et al., 2010; Mäki and Ilmoniemi, 2010; Takemi et al., 2013; Berger et al., 2014) or phosphene sensation (Romei et al., 2008). Importantly, it was also shown that average TMS-electroencephalogram (EEG) responses are reproducible across subjects in case of a stable position of a stimulating coil (Lioumis et al., 2009; Casarotto et al., 2010). In the previous studies, it has been found that the variability of the responses to TMS may be associated with the ongoing brain oscillations (Zarkowski et al., 2006; Sauseng et al., 2009; Mäki and Ilmoniemi, 2010; Dugué et al., 2011; Takemi et al., 2013; Berger et al., 2014). However, the results of these studies were rather heterogeneous: in several early publications a negative correlation between alpha power and the amplitude of MEP during spTMS was reported (Zarkowski et al., 2006; Sauseng et al., 2009). In several other studies no association of the responsiveness to TMS with power in either frequency band was observed (Mäki and Ilmoniemi, 2010; Berger et al., 2014). On the other hand, alternative approaches to probing oscillatory dynamics based on EEG connectivity (Ferreri et al., 2011; Giambattistelli et al., 2014), EEG-EMG coherence analysis (Keil et al., 2014; Schulz et al., 2014) or phase-locking with TMS pulse (Mäki and Ilmoniemi, 2010; Dugué et al., 2011; Berger et al., 2014; Kundu et al., 2014) provided additional insights into the MEP variability.

Compared to rather large number of TMS-EEG studies dedicated to spTMS (Zarkowski et al., 2006; Sauseng et al., 2009; Mäki and Ilmoniemi, 2010; Dugué et al., 2011; Berger et al., 2014), very few were performed with ppTMS (Takemi et al., 2013), yet since short-interval intracortical inhibition (SICI) and intracortical facilitation (ICF) phenomena are known to have primarily intracortical origin (Kujirai et al., 1993; Chen, 2004), they might be more tightly related to cortical oscillations.

Another important question is whether the degree of oscillatory neuronal variability by itself can relate with MEP produced by sp and ppTMS. During the last several years a number of studies specifically focused on the role of brain activity fluctuations and their functional relevance to behavioral and clinical outcomes (Mizuno et al., 2010; Bosl et al., 2011; Hohlefeld et al., 2012; Garrett et al., 2013b; Schlee et al., 2014). The variability of ongoing neuronal activity is thought to reflect intricate synaptic organization of the cortex (Poil et al., 2012; Shew and Plenz, 2013). Moreover, temporal structure of neuronal oscillations is far from being purely stochastic and demonstrates scale-free patterns (Palva et al., 2013). Functional significance of variability in cortical oscillations stems from the findings showing its relationship to behavioral variability (Palva et al., 2013; Smit et al., 2013). Clinically, changes in EEG alpha variability were shown in patients with tinnitus (Schlee et al., 2014) and epilepsy (Larsson et al., 2005) and appeared to be important for the outcome after brain trauma (Hebb et al., 2007). Interestingly, temporal variability in alpha oscillations may be genetically predetermined (Linkenkaer-Hansen et al., 2007) and thus can be associated with the across-subjects variability of the responses to TMS, which is another highly discussed topic in TMS research. This would be in agreement with our recent findings that long-range temporal correlations in the amplitude dynamics of alpha EEG oscillations during rest correlate with the strength of ICF (Fedele et al., 2016). A possibility to use neuronal variability to predict inter-subject difference of the TMS responses is also clinically relevant due to the growing evidence that spTMS and ppTMS phenomena changes may have a diagnostic value in many neurological and psychiatric disorders such as stroke (Bütefisch et al., 2008; Oh et al., 2010; Lioumis et al., 2012), dystonia (Bütefisch et al., 2005; Beck et al., 2009), or schizophrenia (Strube et al., 2014). In this sense particularly interesting is a connection between the variability of TMS responses with respect to the variability of cortical alpha oscillations as they are thought to reflect cortical excitability (Neuper et al., 2006).

Given all the considerations presented above, in the present study we hypothesized that: (1) Not only power but also the variability of alpha oscillations would be predictive of TMS responses. (2) Pre-stimulus alpha oscillations would relate more closely to MEP variability in ppTMS than in spTMS protocols.

## Materials and Methods

### Participants

Seventeen right-handed healthy volunteers (six females) between 19 and 34 years of age (mean: 24 ± 4, SD) participated in the experiment after giving a written informed consent in accordance with the Declaration of Helsinki. Subjects were screened for contraindications to TMS (Rossi et al., 2009) before the consenting process. The experiments were approved by the Local Ethics Committee of National Research University Higher School of Economics, Moscow.

### TMS Coil Positioning and Determination of Threshold

A MagPro X100 (MagVenture) stimulator connected to a water-cooled MCF-B65 induction coil with 75-mm wing radius was used to produce biphasic TMS pulses. A frameless TMS navigation system (Localite TMS Navigator, Localite GmbH) was used for MRI-guided navigation, which ensures consistent coil position and orientation in a 3D space through the sequence of stimulations. TMS coil position was optimized accordingly to individual MR scan (1.5 T MRI scanner; T1 weighted; 1 mm thickness; sagittal orientation; acquisition matrix 256 × 256; MR-scanner Siemens Magnetom Avanto). Stimulation targeted the left primary motor cortex [i.e., motor knob (Yousry et al., 1997)], a “hot spot” for the motor representation of the right abductor pollicis brevis (APB) muscle. The resting motor threshold (RMT) for the given “hot spot” was determined as a minimal stimulator output evoking contralateral APB MEPs of minimum 50 μV in a resting muscle, in 5 out of 10 given stimuli (Rossini et al., 1994).

### Protocol

Three blocks of stimuli (101–114 trials each) were delivered: single-pulse (SP) TMS and two ppTMS protocols: short-interval cortical inhibition (SICI) and ICF. The inter-stimulus intervals (ISIs) for the paired-pulse stimuli were set at 2 ms or 12 ms for SICI and ICF protocols, respectively. The intensity of 110% of RMT was used for spTMS pulses and for the test pulses (S2) in ICF and SICI protocols. Conditioning pulses (S1) for both paired-pulse paradigms had 90% RMT intensity. The interval between the stimuli (or pairs of stimuli) varied randomly from 3 to 10 s and the inter-condition interval varied between 1 to 5 min. We provide the following reasons for determining inter-stimulus intervals. Firstly, we aimed at having a large variability of the intervals in order to avoid the anticipation effect. Secondly, as we used the same distribution for delays in all subjects, the results are unlikely to be due to the chosen inter-stimulus delays but rather would reflect genuine impact of oscillatory activity on cortical excitability. The three blocks were performed in a random order across participants. During all the conditions the coil was held by the operator who constantly monitored its position and orientation with respect to the target using navigation system. Subjects were seated in a comfortable armchair with elbows flexed at 90° and prone hands in a relaxed position, eyes were open and fixed at the mark on the opposite wall.

### EEG and EMG Acquisition

In order to measure EEG, we used BrainAmp DC (Brain Products, Germany) amplifier. 91-electrode BrainCap Fast’n Easy Standard Electrode Cap (Brain Products) was used with TMS-compatible electrodes. The reference electrode was at the bridge of the nose, and ground electrode was placed on the left cheekbone. Three electrooculographic (EOG) electrodes were placed above the nasion and below the outer canthi of the eyes as indicated in (Schlögl et al., 2007). The impedance of each electrode was kept < 5 kΩ throughout the experiment. All data were recorded in the frequency band 0.016–1000 Hz and digitized at a sampling rate of 5 kHz. For the latter analysis the data was re-referenced to a common average electrode.

MEPs to single- and paired-pulse TMS (SP, SICI, ICF) were recorded from the right APB muscle with surface bipolar EMG, using Ag–AgCl bipolar electrodes in a belly–tendon montage and were also recorded with the BrainAmp DC amplifier.

### MEP

EMG activity was high-pass filtered with a fourth order Butterworth high-pass filter (cut-off frequency at 10 Hz) and with the band-stop filter at 50 Hz to remove power-line noise. MEP peaks were identified within the range of 20–62 ms from the onset of the TMS stimulus for the three conditions (SP, SICI, and ICF). This range was sufficient to include MEPs in all subjects. Peak-to-peak measures of the largest positive–negative deflection were used as the MEP amplitudes. Visual inspection was also performed in parallel to remove possible artifactual trials.

### Preprocessing

Electroencephalographic recordings were segmented using TMS event marker. Pre-stimulus segment was 1200 ms (-1210 ms to -10 ms). Channels with excessive amount of noise were excluded from further analysis (maximum 15 channels per subject). Blink-related artifacts in EEG were removed with fastICA algorithm (Hyvärinen, 1999). After the blink removal, we rejected noisy trials according to the variance criteria. For the further analysis we used a pre-stimulus length of 1000 ms for EEG data. The variance of EEG data in each trial was calculated and then a distribution was built. Trials exceeding 99% of the distribution were rejected. Channels with more than 20% artifactual trials were removed (instead of the trials). MEPs corresponding to these trials were excluded from the analysis as well. On average, the percentage of the missing channel was between 15 and 18% for different TMS conditions.

### Power of Pre-stimulus Alpha Oscillations

Pre-stimulus alpha power was estimated from the spectrum calculated with Fast Fourier Transform (FFT), Hanning window (duration 1000-ms immediately preceding TMS pulse in spTMS or conditioning pulse in ppTMS). For the single-trial analysis the power was calculated separately for each pre-stimulus interval. For the across-subjects analysis, the power was averaged across all trials in each condition, electrode and subject. As has been performed in previous studies (Klimesch et al., 1998; Pfurtscheller et al., 2000; Frenkel-Toledo et al., 2013), we considered power in low-alpha (8–10 Hz) and high-alpha (10–12 Hz) sub-bands which might reflect different neuronal processes. Mean substitution method was used for the missing channel values for across subject analysis.

### Variability of Power and MEP Amplitudes

The variability was estimated from single trials both for the amplitude of MEPs and for the pre-stimulus alpha power obtained in each subject, electrode and condition. It was quantified with the coefficient of quartile variation (CQV) (Bonett, 2006) – a descriptive statistic based on quartiles’ information:

(1) $$CQV=\frac{Q_{3}-Q_{1}}{Q_{3}+Q_{1}}$$

In (1), Q_1_ and Q_3_ denote the first (lower) and third (upper) quartiles of the data, respectively. Quartiles are the points that divide any ranked data set into four equal groups. Each group contains a quarter of the data. Q_3_ - Q_1_ is defined as the interquartile range and it is a measure of the spread. Let vectors *P_j_* = *p_1_, p_2_…p_n_* contain pre-stimulus power values from *n* trials in a given subject, condition and *j*th-electrode (*j* = *1,2… E*, where *E* is the number of EEG electrodes). *CQV* will then be applied iteratively to all vectors *P_j_* thus leading to *E* estimates of *CQV*. Similarly, *CQV* was also calculated for the amplitude of MEPs (but only for one bipolar EMG electrode). For each *j*th EEG electrode, CQV of the pre-stimulus power was then correlated with CQV of MEP amplitudes (across subjects) to study the relationship between neuronal variability in the cortex and the variability in motor responses. In general, CQV has an advantage compared to frequently used coefficient of variation since it is less sensitive to the deviations from normality (Bonett, 2006).

### Statistical Analysis

We calculated a repeated measures analysis of variance (ANOVA) in order to compare the CQV of MEP amplitudes among the three TMS conditions. We computed Wilcoxon signed rank test for the comparison of pre-stimulus alpha power among the three TMS conditions. Using the same test, we compared the CQV of pre-stimulus alpha power and CQV of MEP amplitudes between the TMS conditions.

We computed a Spearman correlation of MEP amplitudes between conditions. Besides, we computed a Spearman correlation between alpha power and the amplitude of MEPs for each EEG channel and condition within and across subjects. Within-subject correlations are based on single trial analysis where the power of alpha oscillations is correlated with the corresponding MEP amplitude in each channel, subject, and condition. Moreover, in order to address a relationship between cortical and peripheral variabilities, correlations were computed between the CQV of alpha power versus CQV of MEP amplitudes for each EEG channel and condition across subjects (see above the description of CQV).

In order to take into account multiple comparisons (calculation of tests for many channels), for both Wilcoxon signed rank test and Spearman correlations across subjects, a significance was estimated using cluster-based permutation statistics (Maris and Oostenveld, 2007).

The analysis was performed with custom scripts implemented in Matlab (The MathWorks Inc., Natick, MA, USA).

## Results

### SICI and ICF Strength

**FIGURE 1:**
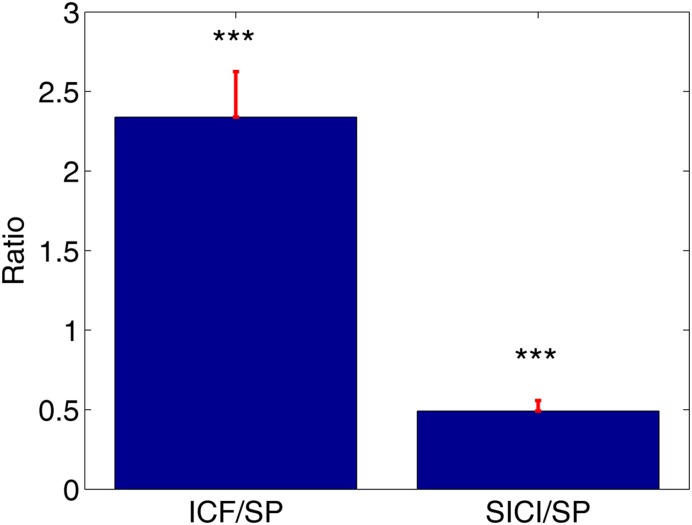
**The average value of the normalized motor evoked potentials (MEPs) with corresponding standard errors.** Intracortical facilitation (ICF)/single-pulse (SP) = 2.34, short-interval intracortical inhibition (SICI)/single-pulse (SP) = 0.49, ^∗∗∗^*P* < 0.001.

ppTMS protocols led to robust SICI and ICF phenomena across subjects. ICF protocol resulted in the increase of MEPs amplitudes compared to MEPs amplitudes during SP condition (ICF/SP mean 2.34 ± 0.29). Likewise, SICI protocol resulted in the significant attenuation of MEPs compared to the SP (SICI/SP mean: 0.49 ± 0.07, **Figure 1**). This was statistically verified with the *t*-tests comparing normalized MEPs against the distribution with unitary mean (^∗∗∗^*P* < 0.001, **Figure 1**).

### Differences between SP, SICI, and ICF Conditions across Subjects

#### Differences in Variability (CQV) of MEPs

**FIGURE 2:**
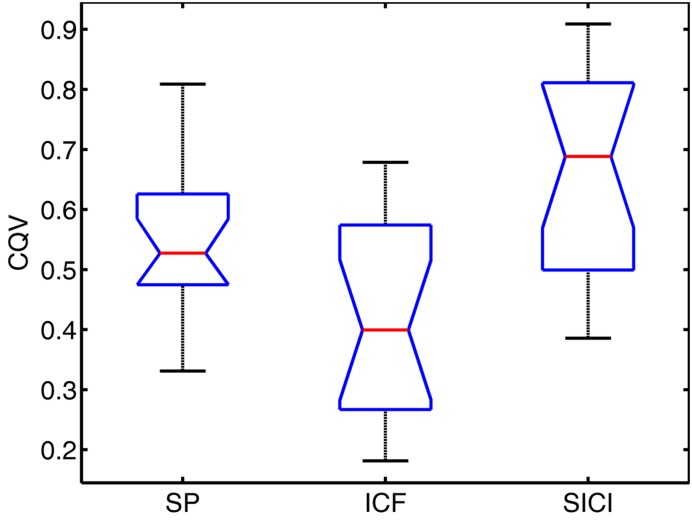
**Notched boxplots for coefficient of quartile variation (CQV) in each TMS condition.** Red lines show the median values. Bottom and top lines of the boxes represent the lower and upper quartiles of the data, respectively. Lower and upper whiskers denote the minimum and maximum CQV values, respectively. Notches show 95% confidence interval of the median value.

The variability of MEPs was quantified with CQV. In **Figure 2**, CQV values of MEP amplitudes across subjects are presented.

Repeated measures ANOVA showed significant differences among conditions (*P* < 0.001). Pairwise comparisons have been performed with Wilcoxon signed-rank test: significantly higher variability of MEPs amplitudes across subjects (assessed with CQV) was observed for SP compared to ICF sessions (*P* = 0.005), SICI had higher CQV compared to SP sessions (*P* = 0.008) and compared to ICF sessions (*P* < 0.002).

CQV of MEP amplitudes between the conditions showed significant positive correlations across subjects between SP and SICI conditions only: SP-ICF (*R* = 0.36, *P* = 0.162); SP-SICI (*R* = 0.64, *P* = 0.007); ICF-SICI (*R* = 0.33, *P* = 0.198).

#### Differences in Pre-stimulus Alpha Power among Conditions

We compared pre-stimulus alpha power between the three conditions using Wilcoxon signed rank test with cluster-based permutation statistics (see Materials and Methods). There were no differences of the alpha power between any of the TMS conditions (SP vs. SICI vs. ICF).

#### Differences in Variability (CQV) of Alpha Power between the Conditions

**FIGURE 3:**
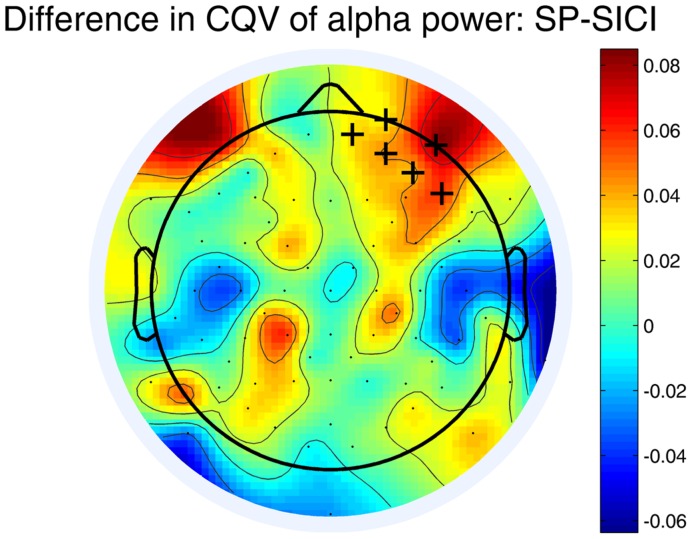
**Differences in CQV of the alpha-power (10–12 Hz) between SP and SICI conditions.** Crosses indicate the channels belonging to a significant cluster (*P* < 0.05) where CQV of alpha power in SP condition is higher. A color shows the difference between the CQVs (SP-SICI).

Variability (CQV) of the alpha power differed between SP and SICI conditions. **Figure 3** shows that the CQV of the alpha power in 10–12 Hz frequency range was significantly higher in SP than in SICI condition (*P* < 0.05). The difference was most pronounced in the right fronto-central area. There were no differences in CQV of alpha power in 8–10 Hz between any of the conditions.

### Correlation between Pre-stimulus Alpha Oscillations and MEPs

#### Correlation between Alpha Power and the Amplitude of MEPs within Subjects

**FIGURE 4:**
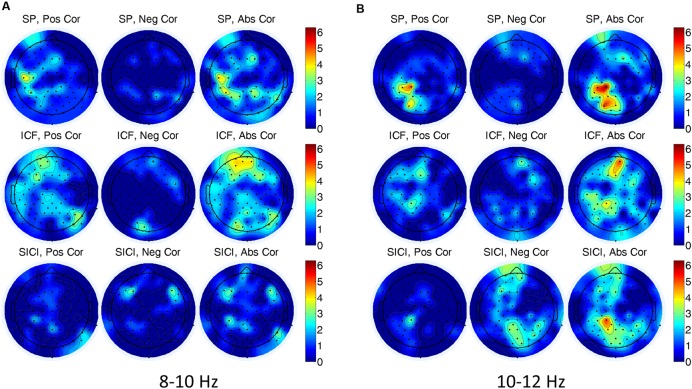
**Number of subjects having significant (*P* < 0.05) correlations between electroencephalogram (EEG) alpha power and the amplitude of MEPs for (A) 8–10 Hz, and (B) 10–12 Hz frequency bands in three TMS conditions.** In **(A,B)** left, center, and right correspond to the number of the subjects for positive, negative, and absolute-value correlations, respectively. A color designates the number of subjects showing a significant correlation.

In single-trial analysis Spearman correlations between EEG alpha power (8–10, 10–12 Hz) and the amplitude of MEPs did not reveal consistently similar channels with significant correlations across subjects. In **Figure 4**, a number of subjects with significant correlations is presented for each EEG channel, TMS condition and alpha band. Note that although there are some scattered clusters of electrodes, the number of subjects with significant correlations at each electrode location is not large, i.e., <5 subjects, (<30%). In addition one can see that the correlations with both signs can be present, thus, not demonstrating a consistent tendency between the oscillatory power and the amplitude of MEPs.

#### Correlation between Alpha Power and the Amplitude of MEPs across Subjects

We computed a Spearman correlation between oscillatory pre-stimulus power and the amplitude of MEPs across subjects using cluster-based permutation statistics. No correlation of the alpha power (averaged across all trials separately for each subject and condition) with the MEP amplitudes across subjects was detected in any TMS condition (SP, SICI, ICF) or in alpha frequency sub-bands. In addition, no correlation of the alpha power was found with the MEPs variability (i.e., CQV of MEP amplitudes during three TMS conditions).

#### Correlation between Variability (CQV) of Alpha Power and Variability (CQV) of MEPs across Subjects

**FIGURE 5:**
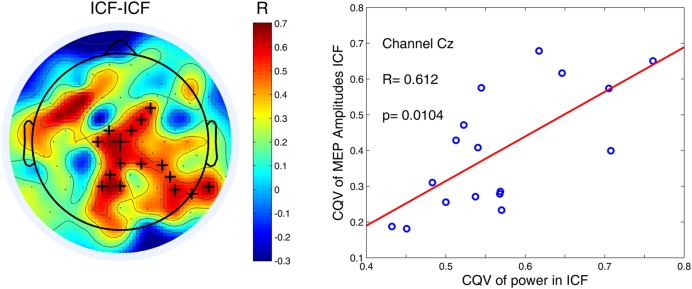
**Variability of high-alpha (10–12 Hz) power in the 1000-ms time window for ICF condition was positively correlated with variability of MEP amplitudes.** Black crosses indicate channels that belong to a significant cluster (*P* < 0.05). An exemplary scatter plot is given for one of the channels (denoted by the largest cross) from a significant cluster.

Finally we assessed a relationship between the variability at both cortical and peripheral levels using cluster-based permutation statistics. Only for ICF condition we found a significant positive correlation between both CQVs (*P* < 0.05). This finding indicates that higher variability of high-alpha (10–12 Hz) power was associated with higher variability of MEP amplitudes (**Figure 5**). As in the case of the other comparisons, there were no significant correlations for low-alpha (8–10 Hz) band in any of the TMS conditions.

Although our main intention for the study was to investigate the relevance of the alpha oscillations for cortical excitability, we also calculated correlations between power of delta (1–3 Hz) and theta (4–7 Hz) oscillations and parameters of MEPs. There were no significant correlations across subjects between power in these bands and MEPs amplitudes or between oscillatory-power CQV and MEP CQV.

## Discussion

There were four main findings of this study. Firstly, we showed that trial-to-trial variability of the MEP amplitudes differed significantly among three TMS conditions. Secondly, we also found a significant difference of trial-to-trial variability of the high-alpha (10–12 Hz) power in the 1000-ms pre-stimulus EEG for SP-SICI comparison (SP > SICI). At the same time, for alpha power no difference among TMS conditions was observed. Thirdly, neither single trial analysis, nor across-subjects approach revealed any significant correlation between pre-stimulus alpha-power and MEP amplitudes. Finally, in the ICF condition larger variability of the high-alpha power in the pre-stimulus EEG was positively correlated with higher variability of MEP amplitudes.

Below we discuss possible explanations of these findings and their significance for the understanding of the within- and across-subject variability of the motor responses in TMS studies.

### The Strength of SICI and ICF across Subjects

Both SICI and ICF were pronounced across subjects but also demonstrated considerable variability, which agrees with previous studies in healthy volunteers (Chen, 2004; Arias et al., 2014; Premoli et al., 2014). Importantly, we found a significant difference in the variability of MEP responses among TMS sessions in the following order: SICI > SP > ICF. A plausible explanation for such difference might be due to the decrease of MEP-amplitude variability with the increase of the MEP amplitude, as previously was shown for spTMS paradigm utilizing different stimulation intensity (Klein-Flügge et al., 2013).

### Trial-to-Trial Variability of Alpha-Power in the Pre-stimulus EEG between TMS Sessions

We have not observed significant differences in the pre-stimulus alpha power across conditions, indicating that there were no changes in the overall generation of alpha oscillations often observed for different experimental conditions including attention (Hanslmayr et al., 2011; Klimesch, 2012; Samaha et al., 2016), sensorimotor performance (Pfurtscheller et al., 1997; Ritter et al., 2009; Babiloni et al., 2014) or differences between normal subjects and patients (Zoon et al., 2013; Schlee et al., 2014). However, we found significant differences between TMS conditions in the pre-stimulus alpha power variability, which was larger in SP compared to SICI condition in the right fronto-central region. The fronto-central alpha was reported to be involved both in emotional (Segrave et al., 2011; Cantisani et al., 2015; Brzezicka et al., 2016) and sensorimotor processes (Chung et al., 2012), which might in our case reflect an induced modulation of the motor system at the high levels by TMS depending on the stimulation condition. Such off-line effects of the prolonged TMS sessions with non-regular inter-stimulus intervals were already reported in several recent studies (Julkunen et al., 2012; Pellicciari et al., 2015; Fedele et al., 2016). Interestingly, the difference of the pre-stimulus EEG alpha power variability was opposite to the direction of the MEP variability, indicating that at least across conditions large variability of the peripheral measures should not necessarily mirror neuronal variability at the cortical level. A possible explanation of the less variable pre-stimulus alpha power during SICI comparing to spTMS condition might be a stabilizing effect of SICI condition activating GABA(A) interneurons (Russmann et al., 2009; Ziemann et al., 2015) circuit, however, such hypothesis is lacking a proof yet. In general, a variability of the brain signals is thought to provide additional information on cortical dynamics when comparing healthy subjects and patients (Nenadovic et al., 2008; Schlee et al., 2014) and for predicting behavioral outcome both at motor (Garrett et al., 2013a,b; Smit et al., 2013) and sensory levels (Misic et al., 2010; Garrett et al., 2013b; Palva et al., 2013).

### Correlation of the Pre-stimulus Alpha-Power and MEP Amplitudes

One of the goals of the present study was to investigate possible relationships between pre-stimulus power of oscillations and MEP amplitudes during prolonged spTMS and ppTMS sessions. Such dependencies are relevant for the development of brain-state triggered stimulation (Walter et al., 2012; Gharabaghi et al., 2014), which can be a new promising therapeutic methodology (Gharabaghi et al., 2014; Karabanov et al., 2016; Zrenner et al., 2016). In this study, we primarily investigated the hypothesis about the inverse relationship between pre-stimulus alpha-power and MEP amplitude, which was proposed in a few previous publications (Zarkowski et al., 2006; Sauseng et al., 2009), but was not supported in several others (Mäki and Ilmoniemi, 2010; Berger et al., 2014). Such connection is consistent with the research in animals demonstrating a correlation between alpha power and firing rate in sensorimotor regions (Haegens et al., 2011) and studies in visual system in humans reporting inhibitory role of alpha band for visual stimuli detection and phosphene threshold (Romei et al., 2008, 2010). Although in some subjects our single-trial analysis revealed significant correlations between pre-stimulus alpha power and MEPs amplitudes, the location of electrodes and the sign of correlation was not consistent when comparing different subjects. In addition, electrodes with significant correlations did not form extended clusters and were rather isolated, indicating a predominantly stochastic character of these correlations. Across-subjects correlations were not significant either, showing that subject-specific levels of alpha activity could not predict MEP amplitude in any of the studied TMS conditions. Considering that such correlation was previously reported only for a small number of subjects: four (Zarkowski et al., 2006) and six (Sauseng et al., 2009), we might claim that our study on 17 healthy volunteers combined with other studies performed on greater number of subjects (Mäki and Ilmoniemi, 2010; Berger et al., 2014) has rather negative support to the hypothesis about the relationship between the pre-stimulus alpha power and MEP amplitudes in the prolonged non-repetitive TMS sessions.

It is interesting to observe that the variability of MEPs on a single trial level is in contrast to reproducible average MEP responses in ppTMS paradigms (Orth et al., 2003; Fleming et al., 2012; Hermsen et al., 2016) and to TMS-evoked EEG responses (Lioumis et al., 2009). Averaging of the single responses (MEPs or TMS-evoked responses) acts as a low-pass filter thus removing neuronal variability on the scale of seconds. Consequently, the average responses reflect rather subjects specific synaptic configuration of the stimulated neuronal networks demonstrating response reproducibility when measurements are performed over the duration of the experiment.

### Variability of Alpha Power Correlates with Variability of MEP Amplitudes in ICF Condition

One of the most remarkable findings of the study is a relationship between two levels of trial-to-trial variability in ICF condition: variability of the high-alpha power over central and right parieto-occipital areas correlated positively with MEP amplitudes variability. Firstly, such a link between central and peripheral levels of the variability corresponds to the previous research connecting brain signal variability with the behavioral variability of both afferent (Boly et al., 2007; Sadaghiani et al., 2009; Palva et al., 2013) and efferent (Smit et al., 2013) processes. Secondly, this finding may be considered as another support for a recently proposed hypothesis that trial-to-trial variability of MEP amplitudes may be by itself an informative measurement of the neuronal state (Conforto et al., 2012). Such hypothesis is in agreement with a well-known role of the variability in the biological systems from the widely used heart rate variability (Chouchou and Desseilles, 2014; França da Silva et al., 2016) to the firing rate variability serving an important role in action preparation (Churchland et al., 2010; Klein-Flügge et al., 2013). A presence of the correlation between central and peripheral variabilities only for ICF condition could be explained by the fact that ICF phenomenon is likely to be based on multiple synaptic connections (Di Lazzaro and Ziemann, 2013; Ziemann et al., 2015). Therefore, it is more spatially distributed in the brain compared to a more local SICI phenomenon (Di Lazzaro et al., 2006). Alpha oscillations, recorded with EEG, also have rather wide spatial distribution and, thus, are more likely to relate to similarly broad ICF neuronal networks than to more spatially specific SICI networks. This observation would also fit recent results, where it was possible to predict ICF but not SP or SICI strength with the EEG neuronal dynamics recorded during rest (Fedele et al., 2016). Moreover, there were no correlations of MEP CQVs in ICF and SP or SICI conditions across subjects which might further support the specificity of a link between cortical and peripheral variability in ICF but not in other conditions.

Interestingly, all our significant results were found for the upper alpha (10–12 Hz) frequency band. In general this agrees with the findings indicating that low (8–10 Hz) and high-alpha (10–12 Hz) sub-bands may be associated with different neuronal processes (Klimesch, 1999; Moore et al., 2008). Thus, low-alpha sub-band may mostly relate to general tonic alertness, while task-specific sensorimotor processes are more associated with high-alpha sub-band (Babiloni et al., 2014).

While we did not find a considerable evidence for the previously reported link between the pre-stimulus EEG alpha power and MEP amplitudes either within-or across-subjects, we were able to demonstrate an importance of a pre-stimulus alpha-power variability for ppTMS phenomena, thus, providing a further support for the hypothesis that ongoing neuronal variability may modulate cortical motor output.

### Limitations of the Study

For both studied ppTMS phenomena only one ISI (2 ms for SICI and 12 for ICF) among several commonly used (Bütefisch et al., 2008; Stagg et al., 2011; Lioumis et al., 2012) was chosen. Importantly, we observed robust ICF and SICI in our subjects, thus making their values suitable for the correlations with the cortical oscillations. Investigation of alternative ISI could be a topic of other studies since each ISI requires a separate EEG experiment due to a large number of the required epochs.

Because of the residual scalp EMG in some of our subjects, we were not able to investigate beta oscillations which are known to be relevant for motor processing. However, our results on alpha rhythm already provide a novel link between the variability in the cortical oscillations and motor responses as tested with ppTMS.

At this stage of the study we did not investigate TMS-EEG responses. Firstly, it was due to our original intention to investigate a relationship between ongoing neuronal oscillations and cortical excitability as probed with MEPs in ppTMS phenomena. Secondly, since our amplifiers did not have a technical possibility to be gated during the TMS pulse, we observed considerable artifacts in the post-stimulus interval thus preventing us from analyzing TMS-evoked responses.

## Author Contributions

ZI processed and analyzed the data, and wrote the manuscript. MN designed the study, collected the data and wrote the manuscript. ZI and MN contributed equally to the study. TF wrote codes for the pre-processing and analysis of the data. EB designed the study and collected the data. VN designed and supervised the study, wrote the manuscript.

## Conflict of Interest Statement

The authors declare that the research was conducted in the absence of any commercial or financial relationships that could be construed as a potential conflict of interest. The reviewer PL declared a past collaboration with one of the authors (MN) to the handling Editor, who ensured that the process met the standards of a fair and objective review.
